# Draft Genome Sequence of Dematiaceous Coelomycete *Pyrenochaeta* sp. Strain UM 256, Isolated from Skin Scraping

**DOI:** 10.1128/genomeA.00158-13

**Published:** 2013-05-30

**Authors:** Su Mei Yew, Chai Ling Chan, Tuck Soon Soo-Hoo, Shiang Ling Na, Seong Siang Ong, Hamimah Hassan, Yun Fong Ngeow, Chee Choong Hoh, Kok Wei Lee, Wai Yan Yee, Kee Peng Ng

**Affiliations:** Tropical Infectious Diseases Research and Education Centre (TIDREC), Department of Medical Microbiology, University Malaya Medical Centre, Faculty of Medicine, University of Malaya, Kuala Lumpur, Malaysiaa; Codon Genomics SB, Jalan Bandar Lapan Belas, Pusat Bandar Puchong, Selangor Darul Ehsan, Malaysiab

## Abstract

*Pyrenochaeta*, classified under the order *Pleosporales*, is known to cause diseases in plants and humans. Here, we report a draft genome sequence of a *Pyrenochaeta* sp. isolated from a skin scraping, with an estimated genome size of 39.4 Mb. Genes associated with the synthesis of proteases, toxins, plant cell wall degradation, and multidrug resistance were found.

## GENOME ANNOUNCEMENT

Fungi belonging to the *Pyrenochaeta* species are dematiaceous coelomycetes that have morphological characteristics similar to those in the *Phoma* species. These fungi have been reported to be important plant pathogens causing root disease in numerous plants, leading to significant plant loss (1, 2). In humans, the diseases caused by *Pyrenochaeta* are mostly onychomycosis and keratitis (3–5). A draft genome sequence of *Pyrenochaeta* sp. strain UM 256 was generated and is reported here to reveal the genes present in *Pyrenochaeta* sp., which are of interest to researchers from different fields. This fungus was isolated from a skin scraping and was identified molecularly using the universal primers ITS1 and ITS4.

Genomic DNA from *Pyrenochaeta* sp. UM 256 was extracted for the generation of a single-end library using the Roche 454 GS FLX+ system and a 3-kb insert size paired-end library using Roche 454 GS Junior. A total of 1,225,229 single-end reads and 199,554 paired-end reads were generated with 27× sequencing depth. The sequence reads were assembled using the GS *de novo* Assembler version 2.70 (Newbler, Roche), and 286 contigs (≥500 bp) were generated. The contigs were then scaffolded into 254 scaffolds (≥1,000 bp) with an N_50_ size of 482 kb. The genome size was estimated to be 39.4 Mb with 50.35% G+C content. GeneMark-ES v2.3e was used to predict 12,545 protein-coding genes with an exon frequency of 2.75 exons per gene from repeat-masked scaffolds (6). The scaffolds were searched against the NCBI Swiss-Prot database, resulting in the annotation of 7,754 (61.81%) genes.

Putative genes of interest found in the UM 256 genome include those involved in the production of proteases, such as aspergillopeptidases and metalloproteinases, which are known to be virulence factors associated with tissue destruction (7); mycotoxins, such as the secondary metabolites gliotoxin, aflatoxin, and sterigamatocystin (8, 9); and enzymes involved in plant cuticle and cell wall degradation, such as cutinase, feruloyl esterase, and glucanase (10, 11). These putative proteins suggest that UM 256 might be a phytopathogenic fungus that is able to invade host tissues and cause immunosuppression. Also found were genes associated with multidrug resistance (*cdr1*, *cdr2*) conferring resistance to azole antifungal agents, and genes encoding proteins resistant to quinidine, flurocytosine, and benomyl/methotrexate, suggesting multidrug resistance (12–17); this is corroborated by the results of *in vitro* antifungal susceptibility tests performed in our laboratory.

### Nucleotide sequence accession numbers.

The nucleotide sequence of the *Pyrenochaeta* sp. UM 256 genome has been deposited in DDBJ/EMBL/GenBank under the accession no. AOUM00000000. The version described in this paper is the first version, accession no. AOUM01000000.
